# Influence of an inspiratory muscle fatigue protocol on healthy youths on respiratory muscle strength and heart rate variability. A randomized controlled trial

**DOI:** 10.3389/fphys.2024.1457019

**Published:** 2024-08-23

**Authors:** Arturo Ladriñán-Maestro, Jorge Sánchez-Infante, Daniel Martín-Vera, Alberto Sánchez-Sierra

**Affiliations:** ^1^ School for Doctoral Studies and Research, Universidad Europea de Madrid, Madrid, Spain; ^2^ Research Group on Exercise Therapy and Functional Rehabilitation, Faculty of Sports Sciences, Universidad Europea de Madrid, Madrid, Spain; ^3^ Faculty of Physiotherapy and Nursing of Toledo, Universidad de Castilla-La Mancha, Toledo, Spain; ^4^ Department of Sport Sciences, Faculty of Sport Sciences, Universidad Europea de Madrid, Villaviciosa de Odón, Spain; ^5^ Physiotherapy Research Group of Toledo (GIFTO), Faculty of Physiotherapy and Nursing, Universidad de Castilla-La Mancha, Toledo, Spain; ^6^ Faculty of Health Sciences, Universidad Francisco de Vitoria, Madrid, Spain; ^7^ Department of Physiotherapy, Faculty of Sport Sciences, Universidad Europea de Madrid, Villaviciosa de Odón, Spain; ^8^ Clínica Sierra Varona SL, Toledo, Spain; ^9^ Department of Physical Therapy, Camilo José Cela University, Madrid, Spain; ^10^ Department of Physical Therapy, Universidad Alfonso X El Sabio, Villanueva de la Cañada, Spain

**Keywords:** inspiratory muscle fatigue, sport, autonomic cardiac control, heart rate variability, respiratory muscle strength

## Abstract

**Introduction:** Inspiratory muscle fatigue has been shown to have effects on the autonomic nervous system and physical condition. This study aimed to evaluate the influence of an inspiratory muscle fatigue protocol on respiratory muscle strength and heart rate variability in healthy youths.

**Materials and Methods:** A randomized controlled clinical trial, employing double-blinding, was conducted with twenty-seven participants aged 18–45 years, non-smokers and engaged in sports activity at least three times a week for a minimum of 1 year. Participants were randomly assigned to three groups: Inspiratory Muscle Fatigue group, Activation group, and Control group. Measurements of heart rate variability, diaphragmatic ultrasound, and maximum inspiratory pressure were taken at two stages: before the intervention and immediately after treatment.

**Results:** In our results with respect to baseline to post-treatment, the inspiratory muscle fatigue group showed lower values in the Sniff contraction velocity variable (10.96 cm/s ± 1.99–8.34 cm/s ± 1.23; *p* < 0.01) and higher values in the activation group (10.59 cm/s ± 0.89–12.66 cm/s ± 1.15; *p* < 0.01) with respect to the control group (10.27 cm/s ± 1.48–9.97 cm/s ± 1.42). On the other hand, the inspiratory muscle fatigue group showed higher values in the Low frequency variable (49.37 n.u. ± 13.91 to 69.48 n.u. ± 8.22; *p* < 0.01) and lower values in the activation group (57.92 n.u. ± 8.37 to 41.59 n.u. ± 11.21; *p* < 0.01) with respect to the control group (50.83 n.u. ± 17.30 to 52.10 n.u. ± 20.64). Additionally, significant correlations were found between respiratory variables and heart rate variability variables.

**Conclusion:** Acute fatigue of the inspiratory musculature appears to negatively impact heart rate variability and inspiratory muscle strength in healthy youths.

**Clinical Trial Registration:** https://clinicaltrials.gov/study/NCT06278714; Identifier: NCT06278714.

## 1 Introduction

Heart rate variability (HRV) quantifies the fluctuations in the time intervals between successive heartbeats, known as RR intervals, as recorded in electrocardiographic or heart rate analyses. This variability reflects the autonomic nervous system’s ability to regulate cardiac function and serves as a crucial indicator of homeostasis and the balance between the sympathetic and parasympathetic systems (Arakaki et al., 2023). Both branches have a significant influence on heart rate. The parasympathetic system, through the release of acetylcholine, decreases heart rate and increases HRV by slowing diastolic depolarization, while the sympathetic system, through the release of epinephrine and norepinephrine, increases heart rate and diastolic depolarization (Wehrwein et al., 2016). HRV is related to various variables such as executive function, decision-making, emotional regulation, and various pathological conditions such as dementia or stroke, among others (Arakaki et al., 2023). In the sports field, HRV is an important marker of exercise-induced fatigue (Ni et al., 2022), allowing for the control of training intensity and determining the adaptations produced by it (Al Haddad et al., 2009; Aubert et al., 2003), HRV can be used as a simple, non-invasive, and validated tool, playing a role in sports programming and recovery, as well as in monitoring internal load (Sanchez-Sanchez et al., 2021).

There is a significant relationship between the respiratory system and the cardiac system, where, for example, both the frequency and depth of breathing affect HRV, producing an increase in HRV and greater parasympathetic activation when breathing is slow and deep, and a decrease in HRV when breathing is rapid and shallow, with a consequent increase in sympathetic activity (Gasior et al., 2016). Regarding the respiratory muscles, particularly the diaphragm, there is also an interaction with the cardiac system, influencing the improvement of venous return, increasing HRV, and decreasing resting and recovery heart rate, due, among other factors, to the diaphragm’s relationship with vagal tone and the parasympathetic nervous system (Kocjan et al., 2017). In contrast, acute respiratory muscle fatigue can limit exercise capacity and have negative cardiovascular effects (Welch et al., 2018). This fatigue increases sympathetic activity, redistributes blood flow to the respiratory muscles, and increases cardiac output associated with these muscles, heightening the perception of effort and decreasing performance and exercise tolerance (Sheel et al., 2018; Romer and Polkey, 1985).

Various studies in the scientific literature have investigated the implication of respiratory muscle fatigue through resistive loads on cardiorespiratory condition (Welch et al., 2018; Smith et al., 2017). However, the analysis of HRV in relation to inspiratory muscle fatigue is poor or nonexistent. The hypothesis of this study is that acute inspiratory muscle fatigue protocol may have a negative effect on inspiratory muscle strength and heart rate variability, while activation of the inspiratory muscles has a positive impact on inspiratory muscle strength and heart rate variability. Consequently, given the multiple correlations and the physiological and athletic importance of this variable, the objective of our study was to objectively evaluate the relationship between acute inspiratory muscle fatigue, inspiratory muscle strength, and HRV.

## 2 Materials and methods

### 2.1 Study design

This study utilized a randomized parallel clinical trial design and was conducted at the Physiotherapy Laboratory of the University of Castilla La Mancha (Toledo, Spain), in accordance with the Consolidated Standards of Reporting Trials (CONSORT) guidelines (Cuschieri, 2019). Informed consent was obtained from all participants. The study was conducted in accordance with the Declaration of Helsinki and approved by the Research Ethics Committee of the Complejo Hospitalario Universitario de Toledo (ID: 1070), and was registered on ClinicalTrials.gov (NCT06278714).

### 2.2 Participants

Using the randomization.com application, twenty-seven young, healthy participants were enlisted in the research and allocated randomly. Three groups of participants were formed: the activation group (AG), the control group (CG), and the inspiratory muscle fatigue group (IMFG). The person who performed this randomization did not take part in the study. The group assignments of the participants were concealed from the assessor and the data analyzer. Individuals between the ages of 18 and 45 who did not smoke and had played sports at least three times a week for a minimum of 1 year met the inclusion criteria. Any medical condition that precluded physical activity, cognitive impairments, chronic diseases (cardiorespiratory, neurological, metabolic, oncological, *etc.*), middle-inner ear pathology or tympanic membrane perforation, lower limb surgery within the previous year, and individuals experiencing an active episode of lower limb pain were among the exclusion criteria. G*Power Software (version 3.1.9.2) was used to determine the sample size based on maximum inspiratory pressure (MIP) ratings that were acquired from a previous investigation (Holtzhausen et al., 2018). Based on a medium effect size (f = 0.25 or partial Eta squared = 0.06) and alpha and beta errors of 0.05 and 0.2, respectively, the computations were performed. Because of the research design, a dropout rate of fourty percent was expected; therefore, a total sample size of twenty-seven participants, split into three groups (n = 9), was needed.

### 2.3 Intervention

Using a threshold valve device (Big Breathe^®^; GH Innotek Co., Ltd., Busan, Republic of Korea), the IMFG undertook a program designed to produce inspiratory muscle fatigue. Subjects breathed against submaximal inspiratory loads of 60% of their maximal inspiratory pressure (MIP) until not being capable to produce airflow in at least three consecutive maximum inspiratory attempts (Welch et al., 2018). Using the same threshold device used by the IMFG, the AG performed two sets of thirty repetitions at 40% of their MIP, in accordance with a previous study (Ozdal, 2016). Conversely, there was no intervention provided to the CG. For 10 min that the intervention and activation groups needed to complete their procedure, the participants in this cohort remained sitting.

### 2.4 Outcomes

MIP measurements, diaphragmatic ultrasound, and heart rate variability assessments were conducted at two different time points: pre-intervention (T1) and immediately post-intervention (T2).

### 2.5 Primary outcomes

#### 2.5.1 Maximal inspiratory pressure

Participants in a sitting posture were assessed for MIP using the MicroRPM^®^ Respiratory Pressure Measurement Device (MicroMedical, United Kingdom). In order to guarantee ventilation through the mouth, nasal passageways were blocked. The participants could only complete up to six maneuvers in a single try, with a 1-minute break in between. Out of three attempts, the highest consistently repeatable result with less than 5% fluctuation was found (Laveneziana et al., 2019).

#### 2.5.2 Diaphragmatic thickness and thickening fraction

**FIGURE 1:**
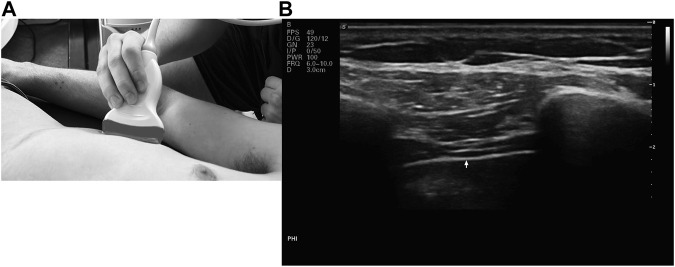
**(A)** Placement of the linear probe on the rib cage **(B)** B-mode ultrasound image of the diaphragm.

The diaphragmatic thickness was measured with an ultrasonic linear probe (L13-3s) operating in the 3.2–12.3 MHz frequency range. The subjects were laying supine, and the probe was placed perpendicular to the chest wall. Measurements were made between the eighth and ninth intercostal gaps at two particular anatomical landmarks: the anterior and mid-axillary lines. In the juxtaposed region, the diaphragm was seen using B-mode ultrasonography (Figures 1A, B). The thickness was measured three times at peak inspiration (Thick_insp_) and end of expiration (Thick_exp),_ and the mean results were noted. The baseline was determined to be the diaphragmatic thickness at the conclusion of expiration. The thickening fraction (TF%) was computed using the following formula: Thickness at the end of maximal inspiration - Thickness at the end of expiration)/Thickness at the end equals TF (Santana et al., 2020).

#### 2.5.3 Diaphragmatic movement curve

**FIGURE 2:**
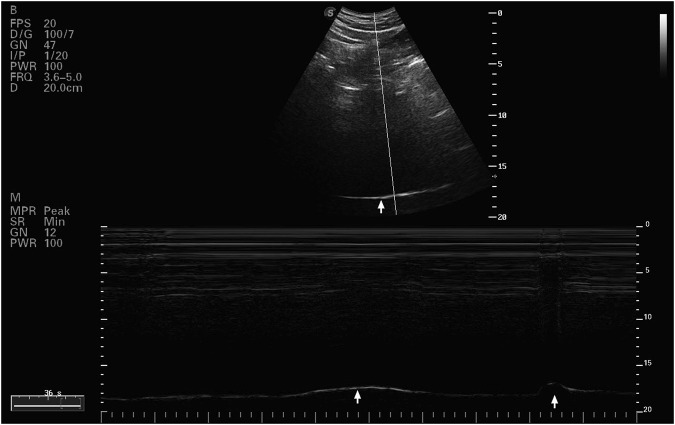
Ultrasound B and M mode image of rigth diaphragmatic dome.

The diaphragmatic movement was evaluated using a convex probe (C5-1s) that ran at 1.2–6 MHz. The liver served as an acoustic window for the probe, which was positioned longitudinally at the right costal margin along the mid-clavicular line. The subjects were lying supine, and the probe was facing the subject’s head. The diaphragmatic movement curve during maximum deep breathing and sniff breathing was recorded using M-mode imaging (Figure 2). For every kind of breathing, parameters such maximal contraction velocity (Vel_insp_ and Vel_sniff),_ inspiratory time (Time_insp_ and Time_sniff_), and diaphragmatic excursion (Mob_insp_ and Mobs_niff_) were examined. The average value for each parameter was collected after three consecutive breathing cycles were analyzed (Santana et al., 2020).

### 2.6 Secondary outcomes

#### 2.6.1 Heart rate variability

A heart rate monitor (Polar H10; Polar Electro Oy, Kempele, Finland) was used to analyze HRV. Participants were placed supine on a stretcher in a quiet room with gentle lighting and a temperature of around 25°C. For 3 minutes, cardiac electrical signals were recorded using a chest band. Participants were told not to consume coffee, alcohol, tobacco, or engage in vigorous physical activity for 12 h prior to the intervention. They were also instructed not to speak and not to move voluntarily while the analysis was being conducted (Schaffarczyk et al., 2022). All measurements were taken during the same time frame, between 9 and 11 a.m. Kubios HRV Analysis Software 3.1.0 for Windows (Biomedical Signal and Medical Imaging Analysis Group, Department of Applied Physics, University of Kuopio, Finland) was utilized for the analysis. Six features were examined: the sympathovagal balance index (LF/HF), power in both low frequency (LF; 0.04–0.15 Hz) and high frequency (HF; 0.15–0.40 Hz), both in normalized units (nu), the standard deviation of all normal-to-normal intervals (SDNN), and the square root of the mean of the sum of squared differences between adjacent normal-to-normal intervals (RMSSD).

### 2.7 Statistical analysis

IBM SPSS Statistics v.22.0 was used for the statistical analysis. A significant criterion of *p* < 0.05 was used. The Kolmogorov-Smirnov test helped to determine each variable’s normality, and the results showed that all of the variables had a normal distribution. To examine the demographic features, descriptive statistics were utilized, and the measurements were reported as mean ± SD. For the outcome variables, a 2-way repeated measures ANOVA was used to analyze the interaction between the Experimental group, Activation group, Control group, and the assessment time (Baseline, Post-treatment). When differences were found, *post hoc* Bonferroni multiple comparisons testing were used. Pearson’s correlation coefficient was used to ascertain the relationship between respiratory variables (MIP, Thick_insp_ and Mob_sniff_) and between HRV variables (RR, SDNN, LF, HF, LF/HF and RMSSD). Cohen’s scale (Cohen, 1969) was utilized to evaluate effect size (ES), which was classified as low (<0.20), medium (0.50), and high (>0.80).

## 3 Results

### 3.1 Demographic data

**FIGURE 3:**
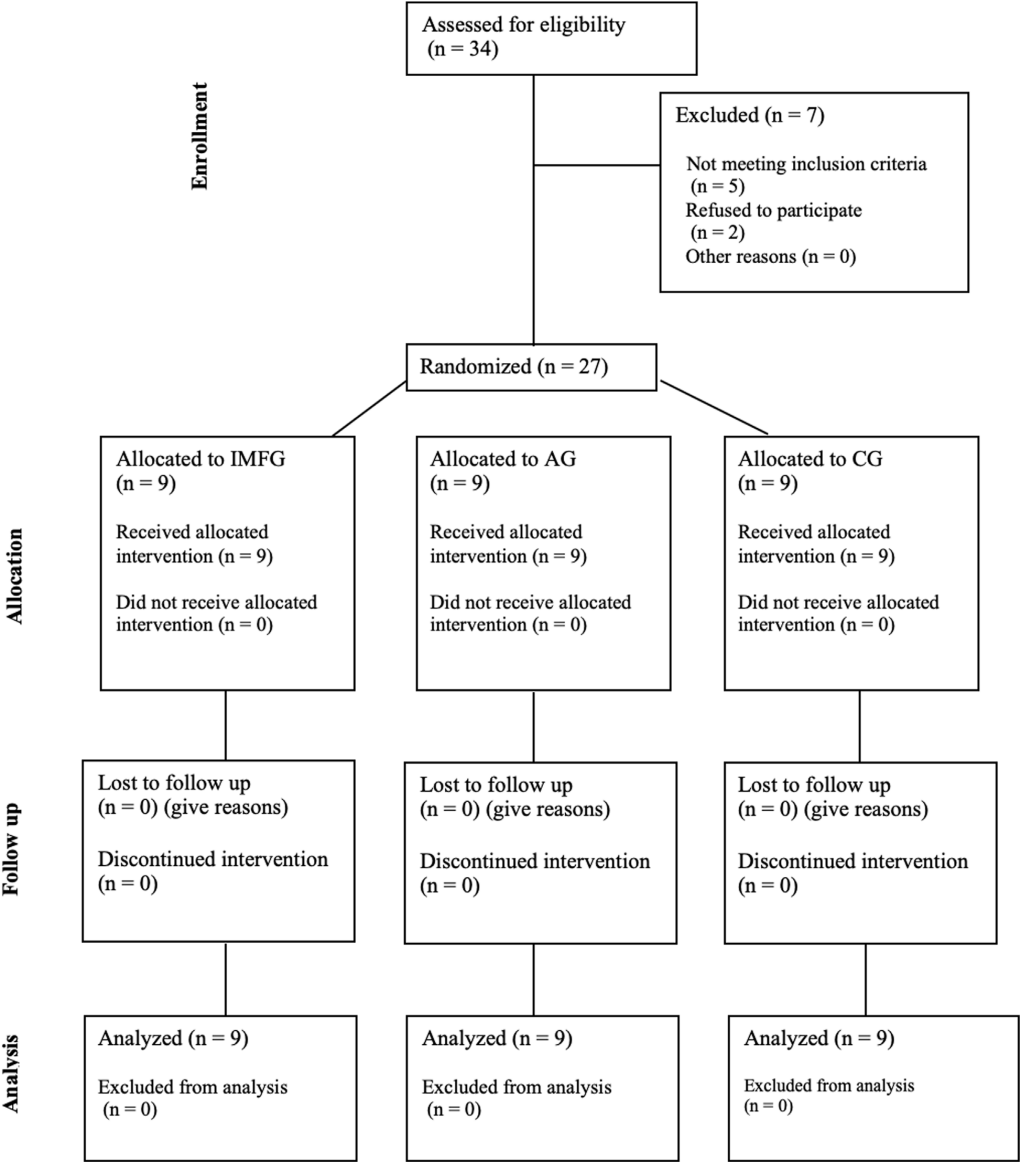
CONSORT flow chart.

Twenty-seven adults were recruited for the study on March 2024 and participate between May and June 2024. They were distributed among IMFG (4-men, 5-women), AG (5-men, 4-women) and CG (4-men, 5-women). There were no dropouts due to the intervention or measurements. The CONSORT flow chart was included (Figure 3). No significant differences were found between IMFG, AG and CG in demographic characteristics (Table 1). Pearson’s correlation revealed a significant association between respiratory variables and HRV variables. The results of this correlation are shown in Table 2.

**TABLE 1:** Demographic characteristics of subject.

	IMFG (n = 9)	AG (n = 9)	CG (n = 9)	p (s)
Sex (male/female)	4/5	5/4	4/5	
Age (yrs)	20.13 ± 2.03	20.38 ± 1.77	20.25 ± 1.49	n.s
Weight (kg)	67.38 ± 6.55	66.63 ± 7.29	66.25 ± 7.96	n.s
Height (cm)	170.50 ± 5.24	169.00 ± 3.55	168.75 ± 5.70	n.s

IMFG, Inspiratory Muscle Fatigue group; AG, Activation group; CG, Control group.

**TABLE 2:** Pearson’s correlation between respiratory variables and between heart rate variability variables.

		RR_Diff_	SDNN_Diff_	LF_Diff_	HF_Diff_	LFHF_Diff_	RMSSD_Diff_
MIP_Diff_	PC	0.932**	0.879**	−0.823**	0.774**	−0.834**	0.879**
	p	0.000	0.000	0.000	0.000	0.000	0.000
Thickinsp_Diff_	PC	0.841**	0.867**	−0.866**	0.825**	−0.897**	0.825**
	p	0.000	0.000	0.000	0.000	0.000	0.000
Mobsniff_Diff_	PC	0.738**	0.779**	−0.661**	0.559**	−0.669**	0.672**
	p	0.000	0.000	0.000	0.002	0.000	0.000

PC, Pearson’s correlation; MIP_Diff_, mean difference of maximal inspiratory pressure; Thickinsp_Diff_, mean difference of Diaphragmatic thickness in inspiration; Mobsniff_Diff_, mean difference of Diaphragmatic mobility sniff; RR_Diff,,_ mean difference of Interval (R-Ri); SDNN_Diff_, mean difference of standard deviation of all normal-to-normal intervals; LF_Diff_, mean difference of Low frequency; HF_Diff_, mean difference of High frequency; LFHF_Diff_, mean difference of the sympathovagal balance index as the ratio between low and high frequency power; RMSSD_Diff_, mean difference of the square root of the mean of the sum of squared differences between adjacent normal-to-normal intervals.

* The correlation is significant at the 0.05 level (bilateral).

** The correlation is significant at the 0.01 level (bilateral).

### 3.2 Changes in respiratory variables

Results for primary outcomes are presented in Table 3.

**TABLE 3:** Outcome measurements of respiratory variables.

	Baseline	Post-treatment		f	p	n^2^	pot
RR (ms)							
IMFG	867.29 ± 79.62	654.48 ± 65.14**^##^	Group	3.70	0.04	0.24	0.62
AG	794.21 ± 69.97	926.26 ± 78.88**^$$^	Time	17.99	<0.01	0.43	0.98
CG	781.95 ± 98.25	772.18 ± 110.24+	Group × Time	198.58	<0.01	0.94	1
SDNN (ms)							
IMFG	48.73 ± 6.92	31.33 ± 8.81**^##^	Group	1.90	0.17	0.14	0.36
AG	42.48 ± 14.27	55.38 ± 13.34**	Time	4.22	0.05	0.15	0.51
CG	48.43 ± 10.38	47.88 ± 10.15+	Group × Time	114.65	<0.01	0.91	1
LF (n.u.)							
IMFG	49.37 ± 13.91	69.48 ± 8.22**^##^	Group	1.81	0.19	0.13	0.34
AG	57.92 ± 8.37	41.59 ± 11.21**	Time	0.20	0.66	<0.01	0.07
CG	49.26 ± 17.46	47.90 ± 20.64+	Group × Time	34.40	<0.01	0.74	1
HF (n.u.)							
IMFG	50.58 ± 13.72	28.63 ± 7.11**^#^	Group	1.69	0.21	0.12	0.32
AG	42.88 ± 13.14	50.63 ± 12.57*	Time	4.84	<0.05	0.17	0.56
CG	50.83 ± 17.30	52.10 ± 20.64^++^	Group × Time	21.15	<0.01	0.64	1
LFHF (n.u.)							
IMFG	1.12 ± 0.60	2.56 ± 0.67**^##^	Group	2.82	0.08	0.19	0.50
AG	1.49 ± 0.57	0.89 ± 0.38**	Time	8.76	<0.01	0.27	0.81
CG	1.20 ± 0.81	1.25 ± 1.00^++^	Group × Time	36.47	<0.01	0.75	1
RMSSD (ms)							
IMFG	41.67 ± 7.83	21.67 ± 4.80**^##^	Group	13.14	<0.01	0.52	0.99
AG	40.57 ± 6.64	52.86 ± 5.64**^$$^	Time	13.11	<0.01	0.35	0.94
CG	43.57 ± 6.01	42.11 ± 9.13^++^	Group × Time	123.08	<0.01	0.91	1

IMFG, inspiratory muscle fatigue group; AG, activation group; CG, control group; RR, Interval (R-Ri); SDNN, standard deviation of all normal-to-normal intervals; LF, low frequency; HG, high frequency; LFHF, the sympathovagal balance index as the ratio between low and high frequency power; RMSSD, the square root of the mean of the sum of squared differences between adjacent normal-to-normal intervals.

Values are mean ± SD.

*P < 0.05, **P < 0.01, post-treatment, with baseline.

^#^ *P* < 0.05, ^##^ *P* < 0.01, comparisons between the IMFG and AG groups at corresponding time points.

^+^ *P* < 0.05, ^++^ *P* < 0.01, comparisons between the IMFG and CG groups at corresponding time points.

^$^ *P* < 0.05, ^$$^ *P* < 0.01, comparisons between the AG and CG groups at corresponding time points.

In the analysis of the MIP variable, the IMFG analysis, there was a decreased between baseline and post-treatment of −8.89 ± 1.54 cmH_2_O (*p* < 0.01; ES = 0.63; 95% CI of the difference = −9.97 to −7.81). In contrast, the AG showed an increased between baseline and post-treatment of 2.89 ± 1.96 cmH_2_O (*p* < 0.01; ES = 0.15; 95% CI of the difference = 1.81–3.97). In the analysis of the Thick_insp_ variable, the IMFG analysis, there was a decreased between baseline and post-treatment of −0.03 ± 0.01 cm (*p* < 0.01; ES = 0.43; 95% CI of the difference = −0.04 to −0.03). In contrast, the AG showed an increased between baseline and post-treatment of 0.02 ± 0.01 cm (*p* < 0.01; ES = 0.24; 95% CI of the difference = 0.00–0.02). In the analysis of the Mob_sniff_ variable, the IMFG had lower values than the CG (*p* < 0.01), and the AG had higher values compared to the CG after performing the treatment (*p* < 0.01). Within the IMFG analysis, there was a decreased between baseline and post-treatment of −1.01 ± 0.21 cm (*p* < 0.01; ES = 2.29; 95% CI of the difference = −1.10 to −0.91). In contrast, the AG showed an increased between baseline and post-treatment of 0.49 ± 0.10 cm (*p* < 0.01; ES = 1.43; 95% CI of the difference = 0.39–0.58).

### 3.3 Changes in heart rate variability

Results for primary outcomes are presented in Table 4.

**TABLE 4:** Outcome measurements of heart rate variability.

MIP (cmH_2_O)							
IMFG	98.56 ± 14.06	89.67 ± 13.60**	Group	0.46	0.65	0.04	0.11
AG	99.78 ± 18.79	102.67 ± 18.66**	Time	40.50	<0.01	0.63	1
CG	97.56 ± 15.13	97.78 ± 15.51	Group × Time	138.95	<0.01	0.92	1
Thick_insp_ (cm)							
IMFG	0.47 ± 0.08	0.43 ± 0.07**	Group	0.39	0.68	0.03	0.11
AG	0.46 ± 0.07	0.48 ± 0.07**	Time	9.67	<0.01	0.29	0.85
CG	0.48 ± 0.08	0.48 ± 0.07	Group × Time	48.45	<0.01	0.80	1
Thick_esp_ (cm)							
IMFG	0.23 ± 0.04	0.25 ± 0.04**	Group	0.18	0.84	0.02	0.08
AG	0.23 ± 0.04	0.23 ± 0.03	Time	13.62	<0.01	0.36	0.94
CG	0.24 ± 0.04	0.24 ± 0.04	Group × Time	8.19	<0.01	0.41	0.94
TF (%)							
IMFG	104.79 ± 9.96	75.87 ± 10.65**^##^	Group	8.04	<0.01	0.40	0.93
AG	104.24 ± 8.03	111.01 ± 10.74*	Time	28.45	<0.01	0.54	1
CG	102.81 ± 11.02	99.39 ± 10.12^++^	Group × Time	44.10	<0.01	0.79	1
Mob_insp_ (cm)							
IMFG	6.50 ± 0.44	5.49 ± 0.49**^##^	Group	10.97	<0.01	0.48	0.98
AG	6.53 ± 0.34	7.02 ± 0.27**^$^	Time	48.17	<0.01	0.67	1
CG	6.57 ± 0.32	6.55 ± 0.33^++^	Group × Time	276.93	<0.01	0.96	1
Time_insp_ (ms)							
IMFG	1646.66 ± 198.68	1747.78 ± 199.80**^##^	Group	5.90	<0.01	0.33	0.83
AG	1568.89 ± 79.60	1453.33 ± 57.88**	Time	0.15	0.70	<0.01	0.07
CG	1538.89 ± 50.36	1542.22 ± 61.19^++^	Group × Time	42.59	<0.01	0.78	1
Vel_insp_ (cm/s)							
IMFG	4.00 ± 0.52	3.18 ± 0.45**^##^	Group	17.53	<0.01	0.59	1
AG	4.18 ± 0.32	4.83 ± 0.12**^$$^	Time	3.24	0.08	0.12	0.41
CG	4.28 ± 0.25	4.25 ± 0.29^++^	Group × Time	159.40	<0.01	0.93	1
Mob_sniff_ (cm)							
IMFG	1.80 ± 0.17	1.67 ± 0.14**^##^	Group	4.76	0.02	0.28	0.74
AG	1.85 ± 0.06	1.95 ± 0.10**^$^	Time	2.25	0.15	0.09	0.30
CG	1.85 ± 0.13	1.82 ± 0.09^+^	Group × Time	22.52	<0.01	0.66	1
Time_sniff_ (ms)							
IMFG	167.78 ± 28.19	202.22 ± 20.48**^##^	Group	9.18	<0.01	0.51	0.95
AG	175.56 ± 14.24	155.56 ± 18.78**^$^	Time	2.26	0.15	0.09	0.30
CG	184.44 ± 32.45	185.56 ± 29.20	Group × Time	21.11	<0.01	0.64	1
Vel_sniff_ (cm/s)							
IMFG	10.96 ± 1.99	8.34 ± 1.23**^##^	Group	5.70	<0.01	0.32	0.82
AG	10.59 ± 0.89	12.66 ± 1.15**^$$^	Time	1.88	0.18	0.07	0.26
CG	10.27 ± 1.48	9.97 ± 1.42+	Group × Time	43.04	<0.01	0.78	1

IMFG, inspiratory muscle fatigue group; AG, activation group; CG, control group; MIP, maximal inspiratory pressure; Thick_insp_, Diaphragmatic thickness in inspiration; Thick_esp_, Expiratory diaphragmatic thickness; TF%, Thickness ratio inspiration/expiration; Mob_insp_, Maximal inspiration diaphragmatic mobility; Time_insp_, Maximum inspiratory contraction time; Vel_insp_, Maximum inspiration contraction velocity; Mob_sniff_, Diaphragmatic mobility sniff; Time_sniff_, Sniff contraction time; Vel_sniff_, Sniff contraction velocity.

Values are mean ± SD.

*P < 0.05, **P < 0.01, post-treatment, with baseline.

^#^ *P* < 0.05, ^##^ *P* < 0.01, comparisons between the IMFG and AG groups at corresponding time points.

^+^ *P* < 0.05, ^++^ *P* < 0.01, comparisons between the IMFG and CG groups at corresponding time points.

^$^ *P* < 0.05, ^$$^ *P* < 0.01, comparisons between the AG and CG groups at corresponding time points.

In the analysis of the LF variable, the IMFG had higher values than the AG and CG after performing the treatment (*p* < 0.05). Within the IMFG analysis, there was a increased between baseline and post-treatment of 20.11% ± 13.64% (*p* < 0.01; ES = 1.45; 95% CI of the difference = 13.67–26.56). In contrast, the AG showed a decreased between baseline and post-treatment of −16.32% ± 7.37% (*p* < 0.01; ES = 1.95; 95% CI of the difference = −22.77 to −9.88). In the analysis of the HF variable, the IMFG had lower values than the AG and CG after performing the treatment (*p* < 0.05). Within the IMFG analysis, there was a increased between baseline and post-treatment of −21.95% ± 13.75% (*p* < 0.01; ES = 1.60; 95% CI of the difference = −28.96 to −14.94). In contrast, the AG showed an increased between baseline and post-treatment of 7.75% ± 9.89% (*p* < 0.05; ES = 0.59; 95% CI of the difference = 0.74–14.75).

## 4 Discussion

The results of this research suggest a decrease in inspiratory muscle strength, diaphragm thickness and mobility, as well as an increase in sympathetic activity in the acute inspiratory muscle fatigue group. Conversely, the results obtained in the AG suggest an improvement in inspiratory muscle strength, diaphragm thickness and mobility, in addition to an increase in parasympathetic activity. Again, it should be noted that the results shown in this study are driven by an acute intervention and therefore aim to demonstrate or help understand the acute responses of inspiratory muscle fatigue and inspiratory muscle activation in relation to HRV.

As previously mentioned, acute inspiratory muscle fatigue can condition exercise capacity and performance through various pathways, including neurological, cardiovascular, and metabolic (Romer and Polkey, 1985), and is more prevalent in clinical populations than in healthy subjects (Matecki et al., 2001). Through fatigue stimulation, it triggers peripheral vasoconstriction induced by an increase in sympathetic reflex activity, producing a redistribution of blood flow from the peripheral muscles to the respiratory muscles to maintain ventilatory function based on the required demand (Sheel et al., 2018). The assessment of respiratory muscle strength can be measured through different tools. Measuring MIP is a validated and evidenced method for evaluating inspiratory muscle strength, using portable pressure meters and maneuvers that are easy to learn and tolerate for patients (Laveneziana et al., 2019; Volianitis et al., 2001), and is correlated with transdiaphragmatic pressure in healthy subject (McCool et al., 1997). A moreover, the improvement of this variable is associated with enhanced athletic performance and is related to variables such as maximum oxygen consumption and lactate (HajGhanbari et al., 2013; Fernandez-Lazaro et al., 2021). As for diaphragmatic ultrasound, it is a non-invasive and validated test for assessing diaphragmatic strength and mobility (Zhou et al., 2023), being a reproducible and effective technique in healthy subjects (Wilches-Luna et al., 2022; Scarlata et al., 2019) and correlated with other respiratory variables such as inspiratory muscle strength and transdiaphragmatic pressure (Koco et al., 2021) as well as with the contractile efficiency of these muscles (Sarwal et al., 2013). Regarding our study, a decrease in MIP and various ultrasound variables can be observed in the IMFG, suggesting that the members of this group achieved a status of inspiratory muscle fatigue. These results are consistent with those obtained in previous studies using similar protocols with threshold devices in subjects without pathology (Welch et al., 2018; Smith et al., 2017).

The autonomic nervous system (ANS), as a component of the central nervous system, plays an important physiological role in maintaining homeostasis and regulating various processes, including cardiac function (Duraes Campos et al., 2018). It has two main branches: the parasympathetic system, which decreases heart rate and increases HRV, and the sympathetic system, which increases heart rate and decreases HRV. HRV is an effective method for assessing the ANS, relating to the parasympathetic system through variables such as HF and RMSSD, to the sympathetic system through LF, and to the sympathetic-vagal balance through LF/HF (Perini and Veicsteinas, 2003). In sports, HRV is increasingly used as a mechanism for monitoring internal load due to its ease of use and validity (Sanchez-Sanchez et al., 2021), allowing for quantification of stress, adaptations produced by training, overtraining, fatigue, and recovery capacity in athletes (Lundstrom et al., 2023). It should be noted that, in many cases, lower HRV values may indicate good fitness, as the body exhibits greater arousal and readiness for physical activity (DeBlauw et al., 2023). However, if the inspiratory muscles are weak or fatigued, this sympathetic activation, through the metaboreflex, may induce negative effects that limit athletic performance by reducing blood flow to the extremities, muscle oxygen saturation, or jump performance, among other factors (St Croix et al., 2000; Ladrinan-Maestro et al., 2024). There is an interaction between the respiratory and cardiac systems known as respiratory sinus arrhythmia (RSA), wherein slower and deeper breaths increase RSA, decrease heart rate, and increase HRV (Denver et al., 2007). Based on our results, there appears to be an acute decrease in parasympathetic activity in the IMFG, evidenced by lower HF and RMSSD values, while sympathetic activity increases, shown by higher LF values and decreased RR interval and SDNN, as well as a significant correlation between respiratory variables and HRV variables. This situation can be hypothesized by the aforementioned metaboreflex and the cardiorespiratory interaction mediated by RSA. Increases in sympathetic predominance in athletes can lead to fatigue and overtraining, as well as hormonal imbalances resulting in higher concentrations of stress substances such as cortisol and catecholamines (Clark and Mach, 2016). These results are consistent with another previous and similar study, where acute inspiratory muscle fatigue had negative cardiac effects, increasing blood pressure and heart rate (Welch et al., 2018). In contrast, in the AG, there is an acute improvement in parasympathetic status with higher HF and RMSSD values, a better sympathetic-vagal balance with increased LF/HF, and a decrease in sympathetic activity through lower LF values. Greater parasympathetic predominance can favor sports recovery processes, reducing cortisol levels, improving sleep, and promoting tissue regeneration and recovery processes, thus leading to better training adaptation (Casanova-Lizon et al., 2022). These results align with previous studies where healthy subjects underwent only one session of inspiratory muscle training, resulting in an acute increased parasympathetic activity and sympathetic-vagal balance (Tanriverdi et al., 2021; Rodrigues et al., 2021).

The results obtained in this study may have several interesting practical applications in the respiratory and sports fields. On one hand, the evidence regarding the consequences of acute inspiratory muscle fatigue in young athletes highlights the importance of training these muscles to avoid this negative impact on the autonomic level through sympathetic hyperactivation. On the other hand, the inclusion of an inspiratory muscle activation protocol seems to have potential benefits when applied before sports activities, due to its effects on respiratory muscle strength and HRV.

This study presents several limitations that should be noted. Firstly, although devices like the one used in this study for HRV measurement are validated, the most objective option for measuring HRV remains the electrocardiogram. Similarly, the most objective measurements of respiratory strength are transdiaphragmatic and esophageal pressures. Secondly, these data should be interpreted with caution as they were obtained through an acute intervention with short-term effects. Lastly, certain aspects that could be relevant were not considered, such as fasting vs. postprandial state, chronic caffeine or alcohol consumption, menstrual cycle in women, and dietary control. Therefore, future studies with larger samples and longitudinal measurements are needed to corroborate these results and gather more information as well as explore the different effects of these interventions on amateur and professional athletes, in order to demonstrate how respiratory muscle fatigue and activation affect performance depending on the level of training, as well as to analyze the differences between men and women. The results of this study may serve as a starting point for future research that provides more evidence about the involvement of the respiratory system in sports performance and the central nervous system.

In conclusion, acute inspiratory muscle fatigue appears to have a negative impact on HRV, increasing sympathetic activity and reducing parasympathetic activity, as well as disrupting the sympathetic-vagal balance. On the other hand, activation of the inspiratory muscles seems to improve the sympathetic-vagal balance, enhance parasympathetic activation, and reduce sympathetic activity in healthy young subjects. Further research is necessary to provide more evidence on this topic.

## Data Availability

The raw data supporting the conclusions of this article will be made available by the authors, without undue reservation.
